# Will a government subsidy increase couples’ further fertility intentions? A real-world study from a large-scale online survey in Eastern China

**DOI:** 10.1093/hropen/hoae055

**Published:** 2024-09-17

**Authors:** Wen-Hong Dong, Xia Wang, Fan Yuan, Lei Wang, Tian-Miao Gu, Bing-Quan Zhu, Jie Shao

**Affiliations:** Department of Child Health Care, Children’s Hospital, Zhejiang University School of Medicine, National Clinical Research Center for Child Health, Hangzhou, China; Department of Child Health Care, Children’s Hospital, Zhejiang University School of Medicine, National Clinical Research Center for Child Health, Hangzhou, China; Department of Child Health Care, Zhejiang Maternal, Child and Reproductive Health Care Center, Hangzhou, China; Department of Child Health Care, Children’s Hospital, Zhejiang University School of Medicine, National Clinical Research Center for Child Health, Hangzhou, China; Department of Child Health Care, Children’s Hospital, Zhejiang University School of Medicine, National Clinical Research Center for Child Health, Hangzhou, China; Department of Child Health Care, Children’s Hospital, Zhejiang University School of Medicine, National Clinical Research Center for Child Health, Hangzhou, China; Department of Child Health Care, Children’s Hospital, Zhejiang University School of Medicine, National Clinical Research Center for Child Health, Hangzhou, China

**Keywords:** fertility intention, pro-natal policy, government subsidy, influencing factors, cost-effectiveness, Eastern China

## Abstract

**STUDY QUESTION:**

How many couples with at least one child under 3 years would like to have another one or more child(ren) in Eastern China and will an in-cash subsidy be conducive to couple’s fertility intentions?

**SUMMARY ANSWER:**

In sum, only 15.1% of respondents had further fertility intentions (FFI) before learning about the subsidy, and the planned in-cash subsidy policy increased respondents’ overall FFI by 8.5%.

**WHAT IS KNOWN ALREADY:**

Fertility has been declining globally and has reached a new low in China. The reasons why the Chinese three-child policy was under-realized, and how couples will react to a planned monthly ¥1000 (€141.2) subsidy policy, are not fully understood.

**STUDY DESIGN, SIZE, DURATION:**

During January and February 2022, a cross-sectional online survey aiming to understand families’ expenses of raising a child under 3 years old, and couples’ FFI, was conducted. During the survey period, 272 510 respondents scanned the QR code. This study reports the findings pertaining to questions on respondents’ sociodemographic characteristics, household factors, FFI, and changes in intention from negative to positive after learning about the planned in-cash subsidy. After exclusion, 144 893 eligible responses were included.

**PARTICIPANTS/MATERIALS, SETTING, METHODS:**

Respondents’ FFI, the effect of a planned ¥1000/month*36 months’ in-cash subsidy (€5083.2 in total) on people with a negative FFI before the subsidy, and potential reasons for persistent negative FFI after learning about the subsidy were collected through an anonymous online survey. Stepwise binary logistic regression models were used to select associated factors. The potential fertility rate change and government costs were estimated. A stratified analysis by current child number and sensitivity analysis were also conducted.

**MAIN RESULTS AND THE ROLE OF CHANCE:**

In sum, 15.7% (22 804/144 893) of respondents were male, 15.1% of respondents reported a positive FFI, and 10.0% (12 288/123 051) without an FFI at first changed their intention after learning about the planned in-cash subsidy policy. For those who still said ‘no FFI’, 46.5%, 20.6%, and 14.7% chose pressure on housing status, expenses on children’s education, and lack of time or energy for caring for another child as their first reasons. FFI was strongest in participants receiving the most financial support from their parents, i.e. grandparents (OR = 1.73, 95% CI = 1.63–1.84 for the >¥100 000/year group), and weakest in those already having two children (OR = 0.23, 95% CI = 0.22–0.24). For those with no FFI before learning about the subsidy policy, respondents with the highest house loan/rent (>¥120 000/year, OR = 1.27, 95% CI = 1.18–1.36) were more likely to change their FFI from ‘No’ to ‘Yes’, and those with the highest household income (>¥300 000/year, OR = 0.65, 95% CI = 0.60–0.71) were least susceptible to the policy. In our study population, about 1843 more births every year and an additional 0.3 children per woman were projected under a conservative estimation. Annual estimated cost at the provincial scale would be ¥817.7 (€115.5) million, about 1.02‰ of the total General Public Budget Revenue in 2022. The findings were generally robust in the stratified analysis and sensitivity analysis.

**LIMITATIONS, REASONS FOR CAUTION:**

Selection bias and information errors may exist in the online survey responses. The large sample size and detailed further analysis were used to minimize such biases.

**WIDER IMPLICATIONS OF THE FINDINGS:**

Fertility intentions in Eastern China are rather low. Policymakers should focus more on financial and childcare burdens for a better realization of the three-child policy, including housing, education and childcare services. An in-cash subsidy, which has never been used in China previously, shows promising potential for increasing FFI. However, the application of such policy should be in line with local conditions for better cost-effectiveness regarding fertility-boosting and fiscal sustainability for the government in the long run.

**STUDY FUNDING/COMPETING INTEREST(S):**

This work was supported by the National Key Research and Development Plan of China (2019YFC0840702). The authors declare no conflict of interests.

**TRIAL REGISTRATION NUMBER:**

N/A.

WHAT DOES THIS MEAN FOR PATIENTS? Fertility rates have been declining globally, including in China. Although multiple pro-natal policies have been implemented, the results have been less than satisfactory. This article used data from a large-scale (N = 144 893) online survey to understand the further fertility intentions in families already having one or two children (with one or more of them under 3 years of age) and the effect of a planned in-cash subsidy on fertility intentions, as well as the factors associated with a positive intention, and a positive change in intention due to the subsidy.

Generally, 15.1% out of 144 893 respondents had a positive further fertility intention before learning about the subsidy policy. Financial support from the grandparents and more childcare support from either the father or the grandparents were positively related to fertility intentions. Meanwhile, being the mother, the mother being the main caregiver, high maternal education, high expenses for house loan/rent and children’s education, and already having two children were all negatively associated with fertility intentions.

After learning about the ‘¥1000/month*36 months (€5083.2 in total)’ planned subsidy policy, an overall additional 8.5% of respondents changed their fertility intentions. Respondents with the highest house loan/rent were more likely to change their intention from ‘No’ to ‘Yes’, and those with the highest household income were least likely to be affected by the policy. For people with negative intentions all along, financial burdens of their house, children’s education costs and little time or energy were the top three first reasons.

In a relatively conservative and practical setting, a planned subsidy policy could bring about 1843 more births in the study population. The annual estimated cost at the provincial scale would be ¥817.7 (€115.5) million, which is basically affordable for local government. In general, an in-cash subsidy shows promising potential in boosting fertility and the financial cost of the subsidy for more second or third child births was affordable for the government.

## Introduction

**Figure 1.**
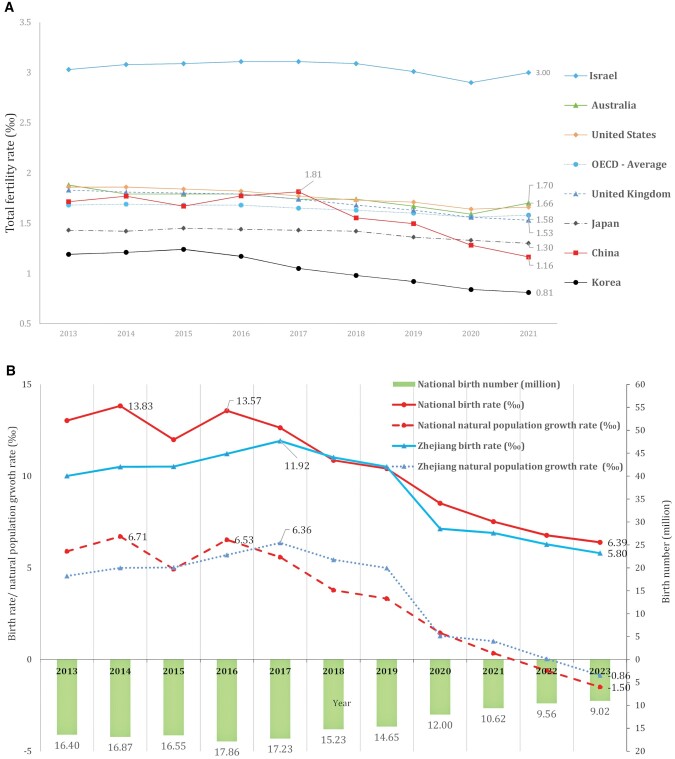
**Birth-related indicators in OECD countries and China during 2013–2023.^a–d^** (**A**) Total fertility rate (‰) in OECD countries and China during 2013–2021. (**B**) Changes in birth-related indicators in China and Zhejiang during 2013–2023. ^a^Total fertility rate data for OECD countries was retrieved from OECD data explorer (https://data-explorer.oecd.org/?lc=en), only 6/38 countries and the average level are presented in (A). Among these, Korea has the lowest rate, and Israel has the highest rate across time. ^b^Data for most OECD countries in 2022 was not available; therefore, only data from 2013 to 2021 was presented. ^c^Fertility data for China was from world bank group (https://www.worldbank.org/en/home); fertility rate for Zhejiang is not available. ^d^All data in (B) was retrieved from China’s National Bureau of Statistics (https://www.stats.gov.cn/).

Since the 1950s, fertility rates have been declining in all countries or territories and more than 53.9% had a total fertility rate below the replacement level (2.1) in 2021, especially in the context of on-going urbanization (Fox *et al.*, 2019). It was projected that in 2100, only six countries would have a fertility rate above the replacement level, and all were located in sub-Saharan Africa (GBD 2021 Fertility and Forecasting Collaborators, 2024). The declining birth rates have multiple impacts on society and on global/regional economies. In China, for example, it was estimated that one-sixth to one-quarter of recent economic growth was contributed by the demographic dividend (Zheng and Rui, 2010); however, it is now compromised due to a continuously declining birth rate (Ye *et al.*, 2021). According to data released by OECD and China’s National Bureau of Statistics (Fig. 1), China’s fertility rate had rebounded and was higher than 84.2% of (32/38) OECD countries in 2017; however, since then, it has been declining and in 2021, it was lower than all OECD countries except for Korea. Meanwhile, the national natural population growth rate, i.e. the difference between the number of births and the number of deaths per 1000 population over a period of time, decreased below zero (−0.60‰) in 2022 for the first time in the last 40 years, and was even lower (−1.50‰) in 2023.

To address this global issue, multiple pro-natal policies, such as abortion restrictions, longer maternity leave with/without basic wages, tax refunding, and child-related cash transfers have been applied by governments around the world (United Nations Department of Economic and Social Affairs, Population Division, 2021). However, the results have been less than satisfactory (Ouedraogo *et al.*, 2018). Some measures such as abortion restriction even showed harmful effects on children, women, and the economy (Pop-Eleches, 2006). For China, pro-natal policies started with the universal relaxation of the one-child policy, which brought 1.99 million more births and a 0.14 increase in national fertility rate during 2016–2017 (Fig. 1). Then, the national Guiding Opinions was issued in 2019, in which three suggestions for promoting childcare services development were proposed: (i) to formulate a multi-dimensional supportive policy and regulation system; (ii) to establish a standard surveillance and assessment system; and (iii) to encourage the development of various forms of childcare services, especially universally affordable services for children <3 years of age (for Zhejiang, this means ≤60% of local monthly per capita disposable income). In August 2021, the universal three-child policy was officially legalized. Then, in March 2022, a ¥1000/month (€141.2/month) childcare tax deduction policy was enacted. Despite all these measures, the birth number, fertility rate and birth rate (birth number per 1000 population) kept declining after the 2016–2017 peak and in 2023, only 9.02 million babies were born, basically half of the number in 2016.

Zhejiang, one of the most developed provinces located in Eastern China, has a tougher situation (Fig. 1B) than the overall situation in China, due to a lower birth rate and longer life expectancy on the basis of a lower all-cause mortality (Zhou *et al.*, 2019). To understand the expenses of raising a child under 3 years old, parents’ further fertility intentions (FFI), obstacles against a fertility rebound, and the potential effect of a planned direct financial subsidy, a cross-sectional online survey aiming at families with one or more child(ren) under 3 years of age was carried out during the 2022 Spring Festival (29 January–9 February 2022). The planned ¥1000/month*36 months subsidy (€5083.2 in total) is a policy that has never been used in China previously and equals 19.9% of the monthly per capita disposable income for people in Zhejiang (32.5% for all Chinese people) in 2022. In total, more than 270 000 parents scanned the quick response (QR) code for the survey.

In the present study, we used data from the above-mentioned large-scale online survey to understand the following factors. (i) How many couples would like to have at least one more child and is there a gender difference in preference? (ii) Which factors would influence respondents’ FFI? (iii) Will the subsidy change respondents’ FFI from negative to positive, who were more affected by the subsidy, and what are the potential effects on fertility rates and cost? Additional data from the survey that were not used in this article are currently being analysed for a separate study.

## Materials and methods

### Study design and population

Briefly, the questionnaire was first designed by the Department of Child Health Care, Children’s Hospital, Zhejiang University School of Medicine, and then revised by an expert panel organized by the Health Commission of Zhejiang Province. In the end, a total of 42 must-answer and 14 conditional must-answer items regarding sociodemographic characteristics, household economic status, fertility, FFI, etc., were included (Supplementary File S1). Since the primary goal of the survey was to understand child-raising expenses for infants and toddlers, two questions were used for targeted population screening in the beginning: (i) Do you have ≥1 child under 3 years? □Yes □No; and (ii) You are the child’s: □Mother □Father or □Grandparents? Only those who chose ‘Yes’ to the first question and ‘Mother/Father’ to the second question were further questioned.

In the formal survey phase, a popular Chinese online platform WJX (https://www.wjx.cn) was used to collect information. The usability and technical functionality of the electronic questionnaire was tested before its official release, and it took about 8–10 min to complete the survey. A unique QR code, an exclusive WJX web-link, and an example survey announcement were first distributed to city-, county-/district- and village-level staff from both Maternal and Children Healthcare Hospitals and regional offices of the Health Commission in all 90 districts/counties in Zhejiang. Then, the print- and/or digital-version QR code was distributed to parents visiting healthcare clinics and in social-media groups so as to reach a more targeted population.

After scanning the QR code, parents were first informed of the aim of the survey, and that they could quit any time before submission; however, once they submit, their answers would be used for policy making. Although anyone with a smartphone could scan the QR code, one could submit their answers only once using the same phone and/or the same QR code-scanning app. Considering the great possibility of replies from different individuals sharing the same internet protocol (IP) address in real life, especially during the Spring Festival, we did not apply IP address restriction (IP restriction means only the first response using a certain IP address is allowed; submissions from others using the same IP address would be rejected). In the survey, respondents could click the ‘back’ button to change or check their answers, and completeness checks were performed using JavaScript before submission. Therefore, submitted questionnaires included those who were not our targeted population and those who had completed all questions.

**Figure 2.**
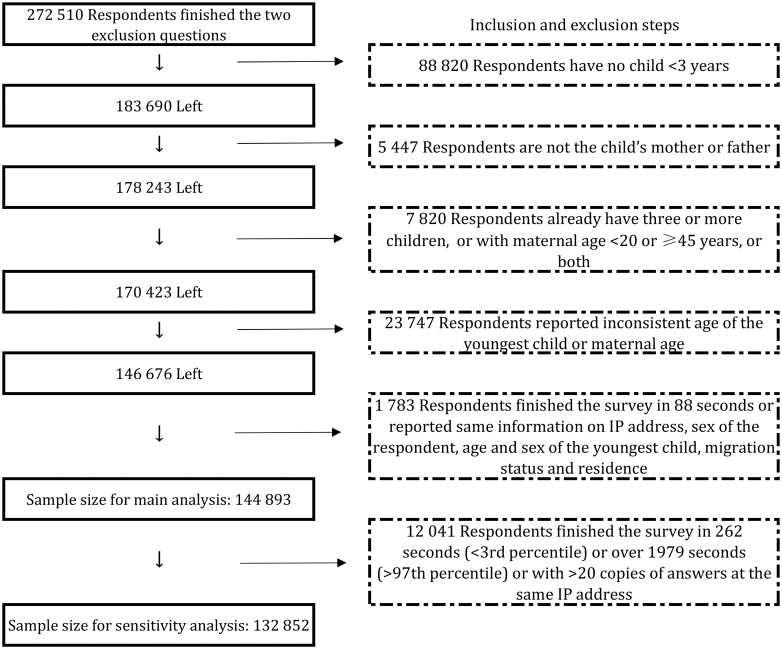
**Respondents’ inclusion and exclusion flowchart**.

In sum, 272 510 respondents answered the two questions for screening and 178 243 mothers/fathers of a child under 3 years submitted full answers. For the present study, we further excluded respondents who already had three or more children, with a maternal age under 20 or above 45 years, with inconsistent answers between items, or if there was high suspicion of duplicate answers from the same person or careless answers (i.e. finishing each item in <2 s) (Bowling *et al.*, 2016) step-by-step. Finally, 144 893 remained in the main analysis (Fig. 2).

### Variables and categories

Sociodemographic characteristics including migration status (local, in-province migrants and inter-province migrants), residence (urban, more developed rural, and under-developed rural), maternal age (5-year intervals from 20 to 45 years), maternal education (≤elementary school, high school, junior college, and university or higher), maternal occupation (farmers and workers, sales and private business owner, teachers, doctors and officers, and houseworkers or otherwise) were inquired in the survey. Family financial status including annual household income (≤100 000, 100 001–200 000, 200 001–300 000 and >300 000 Chinese Yuan, CNY), yearly financial support from grandparents (none, ≤50 000, 50 001–100 000, and >100 000 CNY), annual house mortgage/rent (none, ≤30 000, 30 001–60 000, 60 001–120 000, and >120 000 CNY), and yearly expenses on all children’s education (≤5000, 5001–10 000, 10 001–25 000, and >25 000 CNY) were also collected. We also obtained daily childcare time investment from the mother, the father and the grandparents (0, 0.1–1.9, 2–5.9, and ≥6 h/day). Information on the age, sex and number of children in the family were also collected. The variables used in the survey and definition of the groups are presented in detail in Supplementary File S1.

### Outcomes

Two outcome variables were inquired by asking: (i) Do you intend to have at least one more child? Yes/No. If the answer was No, a conditional must-answer question appeared: (ii) If a ¥1000 (€141.2) monthly in-cash subsidy was provided directly to the family and continues for 3 years after childbirth, will you change your fertility intention from no to yes? Yes/No.

For those still selecting with ‘No’, they were further asked to rank the following potential reasons: (i) great pressure on housing; (ii) high expenses of child-raising; (iii) high expenses of children's education; (iv) short of time or energy for caring for more children; (v) have no trustworthy support/help; (vi) worried about future career development; (vii) disapproved by their partner; (viii) have health condition unfavorable for childbearing; (ix) worried about delivery risks; (x) reasons not listed (with a blank to be filled in). In the analysis, we combined the last five categories as ‘other reasons’ due to low frequencies.

### Statistical analysis

Age of the youngest child was the only continuous variable in our study and described as median (P_25_, P_75_) due to non-normal distribution. All other variables were categorical and presented as N (%). Mann–Whitney *U* tests and Chi-square tests were used for comparing differences between groups. Stepwise logistic regressions were used to understand factors associated with participants’ FFI in the beginning and intention change from ‘No’ to ‘Yes’ due to the in-cash subsidy policy. To better understand the potential effect on fertility and government cost, we performed a crude estimation based on results in our study and publicly available data. Two settings were considered: (i) all FFI was fully realized in 3 years at a constant rate; and (ii) only 50.0% of positive FFI and 5.0% of negative FFI eventually deliver a live birth at a constant rate in the next 3 years (Zhang *et al.*, 2022). Assumptions for estimations and equations are presented in Supplementary Tables S1 and S2. Stratified analyses by current child number were also conducted. *P* values for trends were tested across all subgroups in the final models except for maternal occupation. In a sensitivity analysis for positive FFI and intention change, we further excluded 12 041 responses who answered too fast (<*P*_3_, i.e. 262 s), or too slow (>*P*_97_, i.e. 1979 s), or with >20 responses at the same IP address. In all regression models, ‘Yes’ was treated as the event and ‘No’ as the reference for both targeted outcomes. All analyses were conducted in SAS 9.2 (SAS Institute Inc., Cary, NC, USA), and two-sided *P* values <0.05 were considered significant. We used Excel 2016 (Microsoft, Redmond, WA, USA) and GraphPad Prism 8.0 (GraphPad Software, Boston, MA, USA) to draw figures.

### Ethical approval

The Medical Ethics Committee of Children’s Hospital, Zhejiang University School of Medicine approved this study (No. 2024-IRB-0120-P-01). Since all participants were informed of the aim of this anonymous online survey and a click on the ‘submit’ button denoted their consent for our usage of the information that they submitted, a written consent was waived by the Medical Ethics Committee.

## Results

### Basic characteristics of the respondents

Table 1 presents the basic characteristics of the respondents by sex. Generally, 15.7% (22 804/144 893) respondents were males and the remaining 122 089 (84.3%) were females. Compared with male respondents, females were more likely to be local/rural residents, report maternal occupation as housewives, have two children in the family, have a daughter, have no house loan, and have a higher proportion of maternal childcare ≥6 h/day (all *P *<* *0.0001). Conversely, females were less likely to report an annual household income >200 000 CNY (€28 240), receive any amount of financial support from grandparents, or have ≥6 h/day childcare support from the father (all *P *<* *0.0001).

**Table 1.** Basic characteristics of respondents by sex.

Characteristics	Total	Respondents’ sex		*P*
		Male	Female	
**Total**	144 893 (100.0)	22 804 (15.7)	122 089 (84.3)	–
**Migration status**				**<0.0001**
Local residents	126 235 (87.1)	19 687 (86.3)	106 548 (87.3)	
In-provincial migrants	6440 (4.4)	978 (4.3)	5462 (4.5)	
Inter-provincial migrants	12 218 (8.4)	2139 (9.4)	10 079 (8.3)	
**Residence**				**<0.0001**
Urban	64 261 (44.4)	10 163 (44.6)	54 098 (44.3)	
More developed rural	47 673 (32.9)	7956 (34.9)	39 717 (32.5)	
Rural	32 959 (22.8)	4685 (20.5)	28 274 (23.2)	
**Maternal age, years**				**<0.0001**
20–24	4541 (3.1)	837 (3.7)	3704 (3.0)	
25–29	45 724 (31.6)	7332 (32.2)	38 392 (31.5)	
30–34	63 092 (43.5)	9493 (41.6)	53 599 (43.9)	
35–39	24 902 (17.2)	4046 (17.7)	20 856 (17.1)	
40–44	6634 (4.6)	1096 (4.8)	5538 (4.5)	
**Maternal education level**				**<0.0001**
≤Elementary school	16 148 (11.1)	3003 (13.2)	13 145 (10.8)	
High school	23 645 (16.3)	3683 (16.2)	19 962 (16.4)	
Junior college	40 713 (28.1)	5767 (25.3)	34 946 (28.6)	
University or higher	64 387 (44.4)	10 351 (45.4)	54 036 (44.3)	
**Maternal occupation**				**<0.0001**
Farmers and workers	11 365 (7.8)	2719 (11.9)	8646 (7.1)	
House wives or else	59 968 (41.4)	7772 (34.1)	52 196 (42.8)	
Sales and private business owner	25 183 (17.4)	4213 (18.5)	20 970 (17.2)	
Teachers, doctors and officers	48 377 (33.4)	8100 (35.5)	40 277 (33.0)	
**Current child number**				**<0.0001**
One	73 001 (50.4)	11 640 (51.0)	61 361 (50.3)	
Two	71 892 (49.6)	11 164 (49.0)	60 728 (49.7)	
**Age of the youngest child, months**	17 (8–27)	16 (6–26)	17 (8–27)	**<0.0001**
**Sex of the youngest child**				**0.03**
Boy	74 522 (51.4)	12 209 (53.5)	62 313 (51.0)	
Girl	70 371 (48.6)	10 595 (46.5)	59 776 (49.0)	
**Household income, CNY/year**				**<0.0001**
≤100 000	52 137 (36.0)	7866 (34.5)	44 271 (36.3)	
100 001–200 000	55 803 (38.5)	8669 (38.0)	47 134 (38.6)	
200 001–300 000	21 264 (14.7)	3710 (16.3)	17 554 (14.4)	
>300 000	15 689 (10.8)	2559 (11.2)	13 130 (10.8)	
**Financial support from grand-parents, CNY/year**				**<0.0001**
None	81 463 (56.2)	11 181 (49.0)	70 282 (57.6)	
≤50 000	40 712 (28.1)	7203 (31.6)	33 509 (27.5)	
50 001–100 000	13 882 (9.6)	2609 (11.4)	11 273 (9.2)	
>100 000	8836 (6.1)	1811 (7.9)	7025 (5.8)	
**House loan/rent, CNY/year**				**<0.0001**
None	35 300 (24.4)	4639 (20.3)	30 661 (25.1)	
≤30 000	16 836 (11.6)	2853 (12.5)	13 983 (11.5)	
30 001–60 000	36 185 (25.0)	5915 (25.9)	30 270 (24.8)	
60 001–120 000	37 826 (26.1)	6521 (28.6)	31 305 (25.6)	
>120 000	18 746 (12.9)	2876 (12.6)	15 870 (13.0)	
**Expenses on children’s education, CNY/year**				**0.003**
≤5000	65 438 (45.2)	10 522 (46.1)	54 916 (45.0)	
5001–10 000	23 947 (16.5)	3643 (16.0)	20 304 (16.6)	
10 001–25 000	29 547 (20.4)	4537 (19.9)	25 010 (20.5)	
>25 000	25 961 (17.9)	4102 (18.0)	21 859 (17.9)	
**Childcare by the mother, h/day**				**<0.0001**
0	2108 (1.5)	512 (2.3)	1596 (1.3)	
0.1–1.9	9299 (6.4)	2299 (10.1)	7000 (5.7)	
2–5.9	38 972 (26.9)	7150 (31.4)	31 822 (26.1)	
≥6.0	94 514 (65.2)	12 843 (56.3)	81 671 (66.9)	
**Childcare by the father, h/day**				**<0.0001**
0	9530 (6.6)	1012 (4.4)	8518 (7.0)	
0.1–1.9	52 974 (36.6)	5957 (26.1)	47 017 (38.5)	
2–5.9	52 255 (36.1)	9549 (41.9)	42 706 (35.0)	
≥6.0	30 134 (20.8)	6286 (27.6)	23 848 (19.5)	
**Childcare by the grandparents, h/day**				0.069
0	27 903 (19.3)	4285 (18.8)	23 618 (19.3)	
0.1–1.9	26 910 (18.6)	4223 (18.5)	22 687 (18.6)	
2–5.9	34 159 (23.6)	5510 (24.2)	28 649 (23.5)	
≥6.0	55 921 (38.6)	8786 (38.5)	47 135 (38.6)	

CNY, Chinese Yuan (1 CNY = 0.1412 Euros in 2022). Numbers in bold in the table indicates statistical significance, with *P *<* *0.05. Difference between male and female respondents were compared using Mann–Whitney *U* tests for continuous variables and Chi-square tests for categorical variables.

### FFI prevalence and reasons for persistent negative FFI

Table 2 shows that among all respondents, 15.1% (21 842/144 893) expressed positive FFI in the beginning, and 12 288 of the remaining 123 051 (10.0%) respondents with no FFI at first changed their mind later due to the subsidy policy, leading to an 8.5% (12 288/144 893) absolute increase in the total study population and a 56.3% (12 288/21 842) relative increase compared to respondents with positive FFI before learning about the subsidy policy. For those with a persistent negative intention, 46.5%, 20.6%, and 14.7% chose pressure on housing, high expenses of children’s education, and a lack of time or energy for caring for another child as the first reason for unwillingness, respectively. Compared with male respondents, females had a lower FFI (14.4% vs. 18.6%) and intention change prevalence (9.5% vs. 13.0%), both *P *<* *0.0001. Relative to two-child families, the FFI in one-child families was about 4-times higher (24.0% vs. 6.1%, *P *<* *0.0001). Also, one-child families were slightly more likely to be affected by the subsidy than their counterparts (10.6% vs. 9.5%, *P *<* *0.0001).

**Table 2.** Further fertility intention among all participants and first reason for persistent fertility unwillingness.

Characteristics	All respondents	Respondents’ sex		*P*	Current child number		*P*
		Male	Female		One	Two	
**Total**	144 893 (100.0)	22 804 (15.7)	122 089 (84.3)	–	73 001 (50.4)	71 892 (49.6)	–
**FFI before subsidy**				**<0.0001**			**<0.0001**
No	123 051 (84.9)	18 572 (81.4)	104 479 (85.6)		55 511 (76.0)	67 540 (94.0)	
Yes	21 842 (15.1)	4232 (18.6)	17 610 (14.4)		17 490 (24.0)	4352 (6.1)	
**FFI change after learning about the subsidy policy**							
No	110 763 (90.0)	16 162 (87.0)	94 601 (90.6)	**<0.0001**	49 630 (89.4)	61 133 (90.5)	**<0.0001**
Yes	12 288 (10.0)	2410 (13.0)	9878 (9.5)		5881 (10.6)	6407 (9.5)	
**First reason for persistent negative FFI**							
Pressure on housing status	51 505 (46.5)	8733 (54.0)	42 772 (45.2)	**<0.0001**	23 583 (47.5)	27 922 (45.7)	**<0.0001**
High expenses on children's future education	22 812 (20.6)	2907 (18.0)	19 905 (21.0)	**<0.0001**	9016 (18.2)	13 796 (22.6)	**<0.0001**
Little time or energy for caring for another child	16 263 (14.7)	1920 (11.9)	14 343 (15.2)	**<0.0001**	7590 (15.3)	8673 (14.2)	**<0.0001**
High expenses on child-raising	10 589 (9.6)	1373 (8.5)	9216 (9.7)	**<0.0001**	4790 (9.7)	5799 (9.5)	0.35
Short of trustworthy support	4708 (4.3)	564 (3.5)	4144 (4.4)	**<0.0001**	2403 (4.8)	2305 (3.8)	**<0.0001**
Other reasons	4886 (4.4)	665 (4.1)	4221 (4.5)	**0.0469**	2248 (4.5)	2638 (4.3)	0.08

FFI, further fertility intention. Numbers in bold in the table indicates statistical significance, with *P *<* *0.05. Differences between male and female, one-child and two-child families were compared using Chi-square tests.

### Multivariate analysis for factors associated with FFI and change of intention

In multivariate analysis (Table 3), all interested factors were associated with FFI except for maternal occupation, which was therefore not included in the final model. Apart from classic influencing factors such as rural, inter-provincial migration, advanced maternal age, two existing children, and higher household income, families receiving more financial support (>100 000 CNY/year, >€14 120/year) from grandparents had the highest fertility intention (OR = 1.73, 95% CI = 1.63–1.84) compared with the reference group, as well as those receiving more childcare support from either the father or the grandparents. In addition, families with daughters had a stronger FFI (OR = 1.34, 95% CI = 1.30–1.38). Meanwhile, female respondents, or the mother being the main caregiver, or with university or higher maternal education, were associated with less willingness to have one-more child. Also, respondents who were not free of a house loan/rent or spent more on children’s education were less willing to have more children.

**Table 3.** Factors associated with positive further fertility intention before subsidy and positive intention change after learning about the planned in-cash subsidy policy.**^a^**^,^^b^

Characteristics	Positive FFI before subsidy		Positive FFI change after subsidy	
	OR (95% CI)	*P*_trend_	OR (95% CI)	*P_trend_*
**Sex of the respondent (ref = Male)**	0.77 (0.74–0.80)	**<0.0001**	0.72 (0.68–0.75)	**<0.0001**
**Migration status (ref = Local)**		**<0.0001**		**0.0028**
In-provincial migrants	1.07 (1.00–1.15)		0.94 (0.85–1.03)	
Inter-provincial migrants	1.34 (1.26–1.42)		0.90 (0.84–0.97)	
**Residence (ref = Urban)**		**<0.0001**		**<0.0001**
More developed rural	1.34 (1.30–1.39)		1.06 (1.01–1.10)	
Under-developed rural	1.56 (1.50–1.62)		1.20 (1.15–1.26)	
**Maternal age, years (ref = 20–24)**		**<0.0001**		**<0.0001**
25–29	0.83 (0.77–0.90)		0.81 (0.73–0.90)	
30–34	0.70 (0.65–0.75)		0.70 (0.63–0.78)	
35–39	0.58 (0.54–0.64)		0.74 (0.66–0.82)	
40–44	0.50 (0.44–0.56)		0.72 (0.63–0.83)	
**Maternal education level (ref=≤Elementary school)**		**<0.0001**		**0.0003**
High school	0.82 (0.77–0.87)		1.10 (1.02–1.18)	
Junior college	0.68 (0.64–0.72)		1.02 (0.95–1.09)	
University or higher	0.67 (0.63–0.71)		0.95 (0.88–1.02)	
**Maternal occupation (ref = Farmers and workers)**		–		–
Housewives or else	–		0.98 (0.91–1.05)	
Sales and private business owner	–		0.99 (0.91–1.07)	
Teachers, doctors and officers	–		1.06 (0.98–1.15)	
**Current child number (ref = One)**	0.23 (0.22–0.24)	**<0.0001**	0.92 (0.88–0.96)	**0.0004**
**Age of the youngest child, months**	0.99 (0.99–1.00)	**<0.0001**	0.98 (0.98–0.98)	**<0.0001**
**Sex of the youngest child (ref = Boy)**	1.34 (1.30–1.38)	**<0.0001**	1.08 (1.04–1.12)	**<0.0001**
**Household income, CNY/year (ref =≤ 100** **000)**		**<0.0001**		**<0.0001**
100 001–200 000	0.96 (0.92–0.99)		0.85 (0.82–0.89)	
200 001–300 000	0.97 (0.92–1.02)		0.70 (0.66–0.75)	
>300 000	1.24 (1.17–1.32)		0.65 (0.60–0.71)	
**Financial support from grand-parents, CNY/year (ref = none)**		**<0.0001**		–
≤50 000	1.13 (1.09–1.17)		–	
50 001–100 000	1.41 (1.34–1.48)		–	
>100 000	1.73 (1.63–1.84)		–	
**House loan/rent, CNY/year (ref = none)**		**<0.0001**		**<0.0001**
≤30 000	0.87 (0.82–0.92)		1.16 (1.08–1.24)	
30 001–60 000	0.81 (0.78–0.85)		1.23 (1.16–1.30)	
60 001–120 000	0.83 (0.80–0.87)		1.26 (1.19–1.34)	
>120 000	0.95 (0.90–1.01)		1.27 (1.18–1.36)	
**Expenses on children's education, CNY/year (ref =≤5000)**		**<0.0001**		**0.0091**
5001–10 000	0.90 (0.86–0.94)		1.02 (0.97–1.08)	
10 001–25 000	0.81 (0.78–0.85)		0.92 (0.87–0.97)	
>25 000	0.77 (0.74–0.82)		0.94 (0.88–0.99)	
**Childcare by the mother, hours/day (ref = 0)**		**<0.0001**		–
0.1–1.9	0.88 (0.76–1.02)		–	
2–5.9	0.82 (0.71–0.94)		–	
≥6.0	0.77 (0.67–0.89)		–	
**Childcare by the father, hours/day (ref = 0)**		**<0.0001**		**0.001**
0.1–1.9	1.17 (1.09–1.27)		1.10 (1.01–1.19)	
2–5.9	1.29 (1.20–1.39)		1.09 (1.01–1.18)	
≥6.0	1.35 (1.25–1.45)		1.18 (1.08–1.28)	
**Childcare by the grandparents, hours/day (ref = 0)**		**<0.0001**		–
0.1–1.9	1.12 (1.07–1.19)		–	
2–5.9	1.22 (1.16–1.29)		–	
≥6.0	1.16 (1.11–1.21)		–	

CI, confidence intervals; CNY, Chinese Yuan (1 CNY = 0.1412 Euros in 2022); FFI, further fertility intention; OR, odds ratio. Numbers in bold in the table indicates statistical significance, with *P *<* *0.05. Step-wise logistic regression models were used in understanding associated factors of positive FFI in the beginning and positive FFI change from ‘No’ to ‘Yes’ after learning about the subsidy policy.

a Only factors included in the final model of stepwise logistic regressions were presented.

b In the models, ‘Yes’ was treated as the event and ‘No’ as the reference for each outcome, i.e. OR > 1 denoting a higher likelihood of positive FFI/positive FFI change relative to the reference group.

For participants without FFI in the beginning, all interested factors were associated with intention change except for grandparental financial/childcare support and maternal childcare engagement (Table 3). Respondents with house loan/rent >¥120 000/year (>€16 944/year, OR = 1.27, 95% CI = 1.18–1.36) were most likely to change their intention, while families with a household income >¥300 000/year (>€42 360/year, OR = 0.65, 95% CI = 0.60–0.71) were least likely to be affected by the subsidy policy.

### Estimation of subsidy effect and cost

As presented in Supplementary Table S1, when all intentions were fulfilled in our study population at a constant rate in the next 3 years, the subsidy policy would bring 4096 more additional births annually and the fertility rate would increase by 0.7 child per woman; in a more conservative and practical setting, 1843 more additional births every year and an extra 0.3 child per woman would be due to the subsidy policy. The estimated annual cost at provincial-scale would be 817.7 million CNY (115.5 million Euros), which was about 1.02‰ of the total General Public Budget Revenue in 2022 (GPBR). If the policy was applied in all births regardless of parities, the cost would be 6.42-times that of GPBR (Supplementary Table S2).

### Stratified and sensitivity analysis

**Figure 3.**
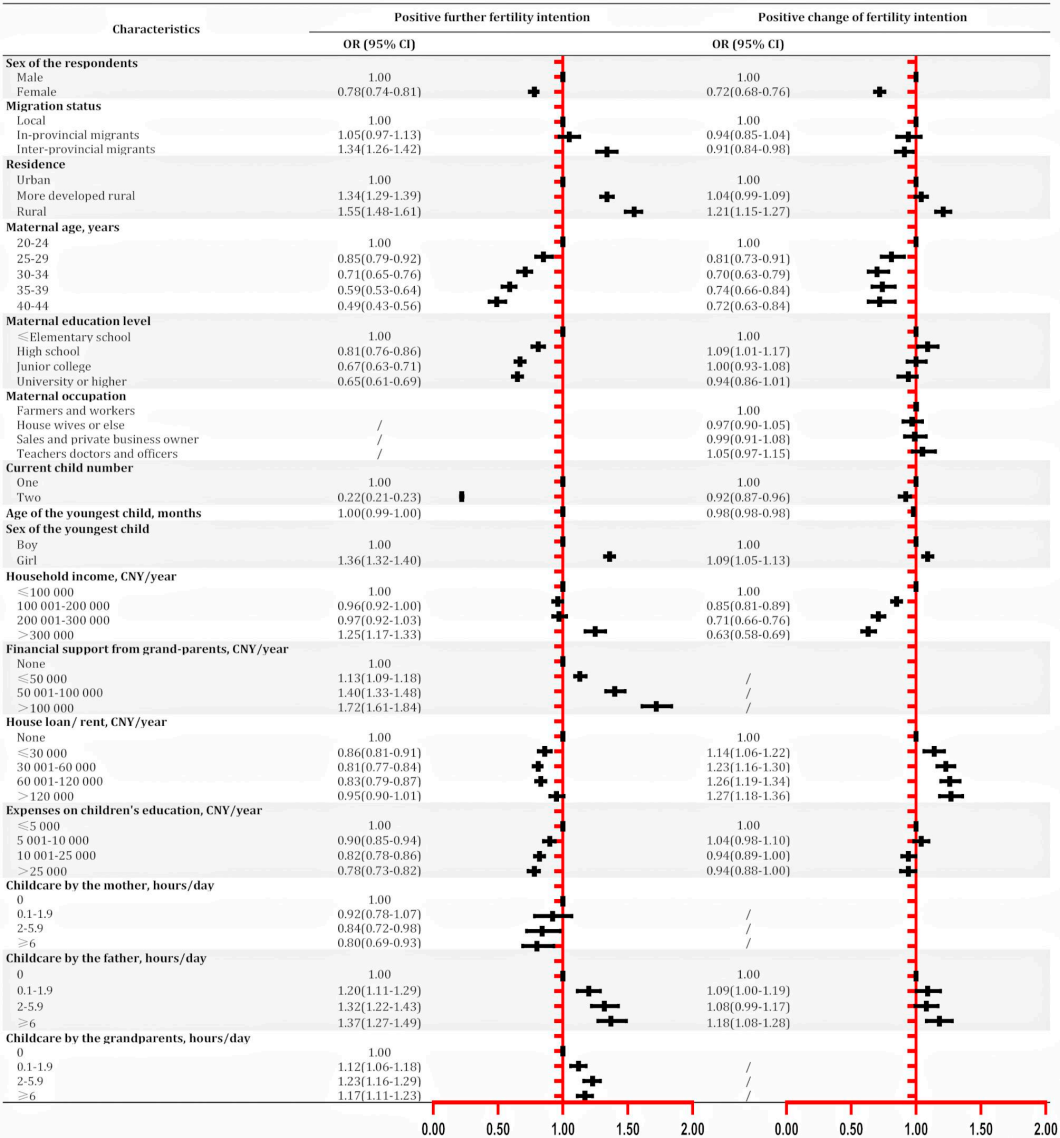
**Factors associated with positive further fertility intention and positive intention change: sensitivity analysis.^a,b^** CNY, Chinese Yuan (1 CNY = 0.1412 Euros in 2022); FFI, further fertility intention; OR, odds ratio; CI, confidence intervals. Step-wise logistic regression models were used to understand factors associated with positive FFI in the beginning and positive FFI change after learning about the subsidy policy. ^a^Respondents who answered too fast (<262 s), or too slow (>1979 s), or with more than 20 responses from the same IP address were excluded from the sensitivity analysis; also, only factors included in the final model of stepwise logistic regressions were presented. ^b^In the models, ‘Yes’ was treated as the event and ‘No’ as the reference for each outcome, i.e. OR>1 denotes a higher likelihood of positive FFI/positive FFI change relative to the reference group.

In a stratified analysis (Supplementary Table S3) by current child number for FFI, findings were similar to that in the main analysis except that the age of the youngest child and house loan/rent status were not associated with FFI in one-child and two-child families, respectively. In a stratified analysis for intention change, one-child families from under-developed rural areas (OR = 1.30, 95% CI = 1.21–1.40) and two-child families with any amount of house loan/rent were most likely to change their intention. On the other hand, household income was negatively associated with intention change in all families. In a sensitivity analysis excluding respondents who answered too fast, or too slow, or with more than 20 responses from the same IP address (Fig. 3), the results remained stable.

## Discussion

In this unprecedented large-scale (N ≥ 140 000) online survey involving only respondents already having 1 or 2 children with at least one of them under 3 years old in Eastern China, we found that only 15.1% participants had positive FFI at first, and males had a higher FFI than females. For participants with no FFI, 10.0% changed their intention after knowing about the planned subsidy policy, leading to an 8.5% absolute increase and a 56.3% relative increase in birth numbers if all intentions were realized. For those with a persistent negative FFI, pressure on housing, the expense of children’s education, and a lack of time/energy for caring for more children were the top three first reasons accountable. In the estimation, under a more conservative and practical setting, the policy would lead to 0.3 more children per woman, and it would cost 1.02‰ of the governments’ GPBR annually. The reasons behind FFI and intention change were multi-dimensional.

To begin with, the positive FFI prevalence in our study was lower than in previous studies (Zhang *et al.*, 2021; Jing *et al.*, 2022; Chen *et al.*, 2023; Yang *et al.*, 2023). A few reasons might be responsible. First, having one child per family has become a social norm after decades (1982–2015) of enforcement of the one-child policy (Qiao *et al.*, 2021). Second, the burden on child-rearing resources such as housing are greater in Zhejiang (Chen *et al.*, 2023). Third, worldwide evidence shows that COVID-19-related concerns regarding maternal health, fetal health and financial stress may aggravate the lower fertility intention (Zhu *et al.*, 2020; Afshari *et al.*, 2022; Golovina *et al.*, 2023), although this impact may not last long. Disparities in study time and population are also responsible. Studies conducted after the three-child policy were more likely to report a higher FFI (Jing *et al.*, 2022; Yang *et al.*, 2023).

Gender difference in FFI was also identified. The more negative attitude among females was partially attributable to concerns on future career development, personal freedom, and financial stability (Chan *et al.*, 2015; Golovina *et al.*, 2023; Xu *et al.*, 2023). As reflected by the gap in childcare time in our study, the traditional value of ‘men in charge outside and women inside’ is still prevailing (Chan *et al.*, 2015). A greater duty in childrearing and an increased number of children leads to a greater wage penalty for mothers. In European countries, the wage gap was 3.6–3.8% (Cukrowska-Torzewska and Matysiak, 2020). In China, it can be as high as 29.1% for females working in private enterprises; meanwhile, the gap for males is insignificant (Xu, 2023). Similar to Asian countries such as Korea (Kim and Lee, 2020), a preference for boys still exists in China. Surname inheritance, elderly care utility, and traditional social values in the evaluation of usefulness of a boy are the main reasons (Shang *et al.*, 2022). The good news is that with the relaxation of birth policy, a declining sex ratio has been observed (Tang and Hou, 2024).

Intergenerational childcare support was found to be meaningful in promoting FFI, but co-residence with grandparents may also backfire (Pink, 2017; Yang *et al.*, 2023). Grandparental support can increase FFI by reducing parenting stress and burnout, but it also brings higher burnout in caring for elders (Fu *et al.*, 2023). Comparatively, support from the father was far more important. After all, a responsible partner and stable relationship are the most important parenthood conditions for females (Chan *et al.*, 2015; Xu *et al.*, 2023); also, the father’s involvement in childcare is beneficial for children’s early development (Rollè *et al.*, 2019).

In general, the subsidy policy showed a conducive effect of boosting FFI in respondents with negative FFI, and it was also fiscally affordable for the government on the basis of our estimations. In western countries, an in-cash bonus has been found to be the most effective measure in fertility-boosting and an increase of 0.2 children per woman has been observed (Luci-Greulich and Thévenon, 2013). Data from the USA revealed that the cash payment policy may bring an 8.1% increase in short-term fertility (Cowan and Douds, 2022), particularly in socio-economically disadvantaged populations. In our study, it may bring 0.3 more children per woman in a relatively conservative estimation (33.5% increase in fertility rate); rural, low-income and those with higher house loan/rent were the people most likely affected. Considering the seemingly unstoppable declining fertility worldwide, in-cash subsidies are a promising measure worth trying. Meanwhile, policymakers should bear in mind that: (i) the effect may be different due to disparities in unemployment status, child-rearing ratios in couples, and childcare burdens across countries (Zhang *et al.*, 2023); (ii) an extension of the policy to childless families could be a great burden to the government (in our estimation, it was 6.4 times of the annual GPBR); (iii) the exact subsidy amount would need to be adjusted continuously for better cost-effectiveness in fertility improvement and financial sustainability for the government in the long run, as Singapore has been doing (Tan, 2023).

The top three first reasons for persistent negative FFI identified in our study are consistent with the findings of others (Pailhé *et al.*, 2018; Zhu *et al.*, 2022). Raising a child in China costs 6.28 times that of per capita GDP, much higher than in Australia (2.08), the USA (4.11) and Japan (4.26) (Liang *et al.*, 2024). According to a recent estimation (Hu *et al.*, 2023), an average of 17.1% of household income was used for education in China and this was even higher (56.8%) for low-income families; for Japan or USA, the proportion is only 1.0–2.0%. Non-educational investments such as housing are also extremely high in China. Data from Numbeo (Numbeo.com, 2024) shows that the property price to income ratio was 29.4 in China in 2024, ranking eighth globally, and was 3.5 times higher than Australia (8.4) and 2.6 times higher than Japan (11.3). The combination of school admission requirements and property ownership further increases this burden (Zhu *et al.*, 2022). Furthermore, center-based childcare services, an effective and often-used pro-natal measure (Wood and Neels, 2019), are still underdeveloped in China. Policymakers should pay more attention to these ‘three great mountains’ on people’s shoulders to boost fertility.

Last but not least, although fertility intention has been widely used in reproduction studies, there is always a gap between intention and actualization. In western countries, the gap ranges from 0.1 to 0.7 births per woman; in rare cases like Poland, actual births are even higher than intentions (Beaujouan and Berghammer, 2021). Similar studies are rare in China; only one found that 50.0% of families showing a positive FFI failed to accomplish their intention eventually (Zhang *et al.*, 2022). Reasons for the gap involve religion, job certainty, residence, age, and couples’ agreement in family size, etc. (Babalola *et al.*, 2017). More studies and practices in narrowing this gap are needed in the future.

Our study mainly has three strengths. First, as the survey was conducted right after the enforcement of the ‘three-child policy’, it provides policymakers with first-hand information on understanding FFI prevalence and potential reasons for the under-realized three-child policy. Second, since the potential effect of a direct financial subsidy on FFI and related cost has never been applied or evaluated in China before, our study was the first exploration on such a topic. Third, the sample size was unprecedentedly large, and much information was surveyed, enabling a detailed analysis.

There are several limitations as well. First, a selection bias might exist due to the nature of convenience sampling in the online survey and due to the fact that FFI in families with no child or children older than 3 years of age were not included. Therefore, the generalizability of our findings should be applied with caution. Second, careless responses and multiple replies by the same person might exist. However, apart from restricting services provided by the WJX platform, we performed a sensitivity analysis, and the findings remained robust. Third, when a planned policy is desirable for the public (Vesely and Klöckner, 2020), more people may choose the choice beneficial to themselves; in our case, this would mean more people would change their FFI from ‘No’ to ‘Yes’ after learning about the cash bonus policy so as to promote policy enforcement, which means that an over-estimation of intention change might exist. Fourth, an estimation of the effect of the subsidy on the fertility rate in the total population is unfeasible due to inaccessible key variables.

## Conclusion

In our study, participants of childbearing age had a rather low FFI regarding a second or third child. We hereby suggest that governments looking for a fertility rebound pay more attention to public well-being matters, especially housing, childcare support and education burdens together with gender equity in childcaring. The ¥1000 (€141.2) monthly subsidy per additional child is generally affordable and shows a positive effect in raising FFI; however, a wider coverage of targeted families should be applied with caution and the exact amount across countries would vary for achieving better cost-effectiveness regarding fertility-boosting and financial sustainability for the governments in the long run.

## Supplementary Material

hoae055_Supplementary_Data

## Data Availability

Datasets generated during and/or analyzed in the present study will be available from the corresponding authors on reasonable request.
