# Supplementary material for: Will a government subsidy increase couples’ further fertility intentions? A real-world study from a large-scale online survey in Eastern China
Source: Hum Reprod Open. 2024 Sep 17;2024(4):hoae055. doi: 10.1093/hropen/hoae055 (PMC11484797; doi:10.1093/hropen/hoae055)
Supplement: hoae055_Supplementary_Data [file hoae055_supplementary_data.zip › HRO-24-0170-R2-SuppFileS1.docx]

**Supplementary File S1**

**Child-raising Expenses Survey Questionnaire for Children <3 Years in Zhejiang^a^**

**Dear parents:**

Happy New Year！

To further understand child-raising expenses for children <3 years and to provide evidence to the government for the formulation of pro-natal polices, we sincerely invite you to participate in this survey. During the survey, you can quit anytime; once you submit your answers, we will assume we have your agreement to use the information you submit to help with policy-making. Thank you for your support!

**Working Panel for Child-raising Expenses Survey**

**for Children <3 Years in Zhejiang**

Jan 28^th^ 2022

| **No.^b^** | **Items** | **Variables used and re-categorized in the present study** |
| --- | --- | --- |
|  | **Screening questions for targeted respondents** |  |
| S1 | S1. **single-choice** Do you have ≥1 child under 3 years?  □Yes [Jump to S2] □No [Jump to the end] | Yes. No re-grouping was applied. |
| S2 | S2. **single-choice** You are the child’s:  □Mother [Jump to A11] □Father [Jump to A11] or □Grandparents [Jump to the end] | Yes. No re-grouping was applied. |
|  | [Formal survey started from here] |  |
|  | **Part A. Basic characteristics** |  |
|  | **Section 1. Basic information of children in the family** |  |
| M1 | A11. **single-choice** The youngest child in the family is ________ months old. [Presented as rolling-down options]  □0 month □1 month □2 months □3 months ….□35 months □36 months | Yes. No re-grouping was applied. |
| M2 | A12. **single-choice** The sex of this child is: □Male □Female | Yes. No re-grouping was applied. |
| M3 | A13. **single-choice** Huji status of this child is:  □Local □Migrants from other cities of Zhejiang □Migrants from other provinces | Yes. No re-grouping was applied. |
| M4 | A14. **single-choice** Currently, your residence is: ________City ________District/County  [Zhejiang has 90 districts/counties administered by 11 municipal cities, and they are presented as rolling-down options. Detail options are not listed here] | Yes. The 90 districts/counties were re-categorized into 3 groups in analysis:  Urban: the 5th digit of the 12-digit administrative code is 1;  More developed rural: the 5th digit is 8;  Under-developed rural: the 5th digit is 2. |
| M5 | A15. **single-choice** Currently, you are raising ________children under 18 years’ old in total.  □1 [Jump to M6] □2[Jump to C1.1] □≥3[Jump to C1.2] | Yes. No re-grouping was applied. |
| C1.1 | A15.1 **single-choice** Age of the older child is:  □Same as the youngest child (twins, triplets, etc.)  □12-24 months □25-36 months □3-6 years □≥6 years’ old  [After answering C1.1, jump to M6] | Not used. |
| C1.2 | A15.1 **matrix single-choice** Age of the other children is:   \| Birth sequence \| Same as the youngest child (twins, triplets, etc.) \| 12-24 months \| 25-36 months \| 3-6 years \| ≥6 years \| Not applicable \| \| --- \| --- \| --- \| --- \| --- \| --- \| --- \| \| 1^st^ \| □ \| □ \| □ \| □ \| □ \| □ \| \| 2^nd^ \| □ \| □ \| □ \| □ \| □ \| □ \| \| 3^rd^ \| □ \| □ \| □ \| □ \| □ \| □ \|   [After answering C1.2, jump to M6] | Not used. |
| M6 | A16. **single-choice** Do you intend to have at least one more child?  □Yes [Jump to M7] □No [Jump to C2] | Yes. No re-grouping was applied. |
| C2 | A16.1 **single-choice** If a ￥1 000 monthly in-cash subsidy was provided directly to the family and continues for 3 years after childbirth, will you change your fertility intention from no to yes?  □Yes [Jump to M7] □No [Jump to C3] | Yes. No re-grouping was applied. |
| C3 | A16.2 **multiple-choice question with ranking** Please choose the reasons for your persistent negative intention to have at least 1 more children and rank them according to their weight in your decision (from the primary reason to the least important reason):  ( ) Great pressure on housing (=1)  ( ) High expenses on child-raising (=2)  ( ) High expenses on children's education (=3)  ( ) Short of time or energy for caring more children (=4)  ( ) Have no trustworthy support/help (=5)  ( ) Worried about future career development (=6)  ( ) Disapproved by the partner (=7)  ( ) Have health condition unfavorable for childbearing (=8)  ( ) Worried about delivery risks (=9)  ( ) Reasons not listed above ___________________________ [One can fill in the blank if they want, the blank is not mandatory] (=10)  [Rank of choices depends on the order they were selected]  [After answering C3, jump to M7] | In analysis, the last 5 options (6-10) were combined as “other reasons” due to low frequencies. |
|  | **Section 2. Basic information of the parents and the family** |  |
| **M7** | **A21.** **matrix single-choice** Age of the child’s father and mother is _________years’ old, respectively.   \| Role \| <20 \| 20-24 \| 25-29 \| 30-34 \| 35-39 \| 40-44 \| 45-50 \| ≥50 \| \| --- \| --- \| --- \| --- \| --- \| --- \| --- \| --- \| --- \| \| Father \| □ \| □ \| □ \| □ \| □ \| □ \| □ \| □ \| \| Mother \| □ \| □ \| □ \| □ \| □ \| □ \| □ \| □ \| | Yes. No change was made in analysis |
| **M8** | **A22.** **single-choice** The child's father's educational level is _________.  □No formal education □Primary school □Elementary school □High school  □Junior college □University (with Bachelor degree)  □University (with Postgraduate/PhD degree) | Not used. |
| **M9** | **A23.** **single-choice** The child's mother's educational level is _________.  □No formal education (=1) □Primary school (=2) □Elementary school (=3)  □High school (=4) □Junior college (=5)  □University (with Bachelor degree) (=6)  □University (with Postgraduate/PhD degree) (=7) | Yes. In analysis, 4 groups were generated:  ≤Elementary school (=1+2+3), High school (=4), Junior college (=5), and University or higher (=6+7). |
| **M10** | **A24.** **single-choice** The father’s occupation is:  □Farmers □Administrative officers  □Professionals such as teachers, doctors/professors □Sales  □Workers □Private business owner □Police officer/soldiers  □Full-time dad □Other job not stated above  □Unemployed/laid-off □Retiree | Not used. |
| **M11** | **A25.** **single-choice** The mother’s occupation is:  □Farmers (=1) □Administrative officers (=2)  □Professionals such as teachers, doctors/professors (=3) □Sales (=4)  □Workers (=5) □Private business owner (=6) □Police officer/soldiers (=7)  □Full-time mom (=8) □Other job not stated above (=9)  □Unemployed/ laid-off (=10) □Retiree (=11) | Yes. In analysis, 4 groups were generated: farmers and workers (=1+5), sales and private business owner (=4+6), teachers, doctors and officers (=2+3+7), and house work or else (=8+9+10).  *Retirees (=11) were excluded before analysis. |
| **M12** | **A26.** **multiple-choice** Apart from the youngest child, who else lives together with him/her:  □Father □Mother  □Grandparents (on the father’s side) □Grandparents (on the mother’s side)  □Elder-brother(s) □Elder-sisters(s) □Younger-brothers(s) □Younger-sister(s)  □Babysitter(s) □Else ___________________________ | Not used. |
|  | **Part B. Household income and expenses** |  |
| **M13** | B1. **single-choice** The annual household income was ________Chinese Yuan in 2021.  □None (=1) □~50 000 (=2) □~100 000 (=3) □~150 000 (=4) □200 000 (=5)  □~300 000 (=6) □~500 000 (=7) □~1 000 000 (=8) □>1 000 000(=9) | Yes. In analysis, 4 groups (CNY) were generated:  ≤100 000 (1+2+3), 100 001-200 000 (=4+5), 200 001-300 000 (=6) and >300 000 (7+8+9) . |
| **M14** | B2. **single-choice** The total financial support from grandparents was ________Chinese Yuan in 2021.  □None (=1) □~50 000 (=2) □~100 000 (=3) □~150 000 (=4) □200 000 (=5)  □~300 000 (=6) □~500 000 (=7) □~1 000 000 (=8) □>1 000 000(=9) | Yes. In analysis, 4 groups (CNY) were generated: none (=1), ≤50 000 (=2), 50 001~100 000 (=3), and >100 000 (=4+5+6+7+8+9). |
| **M15** | B3. **single-choice** The monthly housing loan/rent was ________Chinese Yuan in 2021.  □None (=1) □~2500 (=2) □~5000 (=3) □~7500 (=4) □~10 000 (=5)  □~15 000 (=6) □~20 000 (=7) □>20 000 (=8) | Yes. In analysis, we transferred monthly expense to annual expense (CNY) by multiply the choice by 12 months and 5 categories were generated: none (=1), ≤30 000 (=2), 30 001-60 000 (=3), 60 001-120 000 (=4+5),＞120 000 (6+7+8). |
| **M16** | B4. **single-choice** The monthly expenses used for daily life (cloths, food, water, electricity, gas, etc.) was ________ Chinese Yuan in 2021.  □≤500 □~2500 □~5000 □~10 000 □>10 000 | Not used. |
| **M17** | B5. *single-choice* The annual expenses used for all children’s education was ________ Chinese Yuan in 2021.  □None (=1) □~2500 (=2) □~5000 (=3) □~10 000 (=4) □~25 000 (=5)  □~50 000 (=6) □~100 000 (=7) □>100 000 (=8) | Yes. In analysis, 4 groups of annual total education expenses for all children (CNY) were generated: ≤5000 (1+2+3), 5001-10 000 (=4), 10 001-25 000 (=5), and >25 000 (=6+7+8). |
| **M18** | B6. **single-choice** The annual expenses on medicine/medical treatment for all family members was ________ Chinese Yuan in 2021.  □None □~2500 □~5000 □~10 000 □~25 000 □~50 000 □~10 000 □>10 000 | Not used. |
| **M19** | B7. **single-choice** The annual expenses on insurance for all family members was ________ Chinese Yuan in 2021.  □None □~2500 □~5000 □~10 000 □~25 000 □~50 000 □~10 000 □>10 000 | Not used. |
| **M20** | B8. **single-choice** The annual expenses on taking care of the old was ________ Chinese Yuan in 2021.  □None □~2500 □~5000 □~10 000 □~25 000 □~50 000 □~10 000 □>10 000 | Not used. |
| **M21** | B9. **single-choice** The annual expenses on entertainment such as travelling around was ________ Chinese Yuan in 2021.  □None □~2500 □~5000 □~10 000 □~25 000 □~50 000 □~10 000 □>10 000 | Not used. |
|  | **Part C. Expenses on raising the youngest child <3 years’ old** |  |
|  | **Section 1. Expenses on daily necessities for the youngest child <3 years** |  |
| **M22** | C11. **single-choice** The average monthly expenses on this child’s food such as formula, milk, complementary food, etc. was ________ Chinese Yuan in 2021.  □None □~500 □~1000 □~2500 □>2500 | Not used. |
| **M23** | C12. **single-choice** The average monthly expenses on this child’s dietary supplements such as vitamin D, fish oil, calcium, etc. was ________ Chinese Yuan in 2021.  □None □~100 □~300 □~500 □>500 | Not used. |
| **M24** | C13. **single-choice** The average monthly expenses on this child’s daily consumables such as clothes, shoes, diapers, etc. was ________ Chinese Yuan in 2021.  □None □~500 □~1000 □~2500 □>2500 | Not used. |
| **M25** | C14. **single-choice** The annual expenses on devices/furniture such as baby food processor, sterilizer, baby’s strollers, crib, etc. was ________ Chinese Yuan in 2021.  □None □~500 □~1000 □~2500 □~5000 □~10 000 □>10 000 | Not used. |
|  | **Section 2. Expenses on education for the youngest child <3 years** |  |
| **M26** | C21. **single-choice** Did the youngest child take any parent-child education programs or classes on learning language, music or art in 2021?  □Yes [Jump to C4] □No [Jump to M27] | Not used. |
| **C4** | C21.1 **multiple-choice** The class(es) he/she took included:  □Parent-child class □Second-language class □Music □Art □Sports □Others  [After answering this question, jump to C5] | Not used. |
| **C5** | C21.2 **single-choice** You have spent _______Chinese Yuan in total for all the classes you chose above in 2021.  □≤2500 □~5000 □~10 000 □~25 000 □>25 000  [After answering this question, jump to M27] | Not used. |
| **M27** | C22. **single-choice** You have spent _______Chinese Yuan in total for this child’s books and toys in 2021.  □None □~500 □~1000 □~2500 □~5000 □>5000 | Not used. |
| **M28** | C23. **single-choice** You have spent _______Chinese Yuan in total for this child’s entertainment activities such as playground-playing, zoo-visiting, etc. in 2021.  □None □~500 □~1000 □~2500 □~5000 □>5000 | Not used. |
| **M29** | C24. **single-choice** Did you or other adults in the family take any parenting classes in 2021?  □Yes [Jump to C6] □No [Jump to M30] | Not used. |
| **C6** | C24.1 **single-choice** You have spent _______Chinese Yuan in total for this class.  □None □~500 □~1000 □~2500 □~5000 □>5000  [After answering this question, jump to M30] | Not used. |
|  | **Section 3. Expenses on insurance and medicine for the youngest child <3 years** |  |
| **M30** | C31. **single-choice** You have spent _______Chinese Yuan in pre-pregnancy health check-ups and delivery for this child:  □None □~2500 □~5000 □~10 000 □~50 000 □>50 000 | Not used. |
| **M31** | C32. **single-choice** You have spent _______Chinese Yuan in this child’s vaccination in 2021:  □None □~1000 □~2500 □~5000 □>5000 | Not used. |
| **M32** | C33. **single-choice** You have spent _______Chinese Yuan in health check-ups for this child in 2021:  □None □~1000 □~2500 □~5000 □>5000 | Not used. |
| **M33** | C34. **single-choice** You have spent _______Chinese Yuan in treating diseases of this child in 2021:  □None [Jump to M34]  □~1000 [Jump to C7] □~2500 [Jump to C7] □~5000 [Jump to C7]  □~10 000 [Jump to C7] □>10 000 [Jump to C7] | Not used. |
| **C7** | C34.1 **single-choice** When this child is sick, you normally went to _______for medical services in 2021.  □Pharmacy store □Community-based medical center □County-level maternal and children’s hospital  □County-level comprehensive hospitals □County-level traditional Chinese medicine hospitals  □City-level medical institutions □Provincial-level medical institutions in Zhejiang  □Medical institutions in other provinces | Not used. |
| **M34** | C35. **single-choice** You have bought at least 1 piece of health insurance for this child in 2021:  □Yes [Jump to C8] □No [Jump to M35] | Not used. |
| **C8** | C35.1 **single-choice** The health insurance was:  □ Basic medical insurance system for urban residents  □ Basic medical insurance system for rural residents  □ Commercial insurance  □ Other insurance note listed above  [After answering this question, jump to C9] | Not used. |
| **C9** | C35.2 **single-choice** You have spent _______Chinese Yuan in total for this insurance.  □None □~1000 □~2500 □~5000 □~10 000 □>10 000  [After answering this question, jump to M35] | Not used. |
|  | **Section 4. Expenses on childcare for the youngest child <3 years** |  |
| **M35** | C41. **single-choice** In the first 6 weeks after this child’s birth, the mother and the child were taken care by:  □Grandparents □Paid help □Postpartum care specialist □Postpartum care center  □Grandparents and paid help □Grandparents and postpartum care specialist  □No additional help | Not used. |
| **M36** | C42. **single-choice** You have spent _______Chinese Yuan in total for the above-mentioned support/help.  □None □~5000 □~10 000 □~25 000 □~50 000 □~100 000 □>100 000 | Not used. |
| **M37** | C43. **matrix single-choice** In the last week, how many hours per day on average did the child’s mother, father, or grandparents, etc., spend in taking care of this child?   \| Role \| 0  (=1) \| <1  (=2) \| 1~1.9  (=3) \| 2~3.9  (=4) \| 4~4.9  (=5) \| 6~7.9  (=6) \| ≥8  (=7) \| \| --- \| --- \| --- \| --- \| --- \| --- \| --- \| --- \| \| Father \| □ \| □ \| □ \| □ \| □ \| □ \| □ \| \| Mother \| □ \| □ \| □ \| □ \| □ \| □ \| □ \| \| Grandparents \| □ \| □ \| □ \| □ \| □ \| □ \| □ \| \| Help/Else \| □ \| □ \| □ \| □ \| □ \| □ \| □ \| | Yes. In analysis, 4 groups of childcare support (hours/day) from family members were generated, respectively. They are: 0 (=1), 0.1-1.9 (=2+3), 2- 5.9(=4+5), and ≥6 (=6+7). |
| **M38** | C44. **single-choice** Do you need to pay for the above-mentioned childcare support from the grandparents or the help?  □Yes [Jump to C10] □No [Jump to M39] | Not used. |
| **C10** | C44.1 **single-choice** Last month, you have spent _______Chinese Yuan in total for the support.  □≤2500 □~5000 □~8000 □~10 000 □>10 000  [After answering this question, jump to M39] | Not used. |
| **M39** | C45. **single-choice** Has the child been sent to center-based childcare center?  □Yes [Jump to C11.1]  □No, but we are planning [Jump to C11.2]  □No, and we have no plan to do so in the future [Jump to C12.3] | Not used. |
| **C11.1** | C45.1 **single-choice** The center-based childcare center is:  □Public-owned □Private-owned □Owned by the parents’ employer  □Institutions providing early childhood education □Else__________________  [After answering this question, jump to C12.1] | Not used. |
| **C12.1** | C45.2 **single-choice** Generally, your satisfaction to their childcare service is:  □Not satisfied [Jump to C13.1]  □Basically satisfied [Jump to C14.1]  □Satisfied very much [Jump to C14.1] | Not used. |
| **C13.1** | C45.3 **ranking** Please rank the following reasons for your dissatisfaction:  ( ) High childcare fees  ( ) Suboptimal/lower than expected childcare concept  ( ) Poor quality of the staff  ( ) Far distance  ( ) Less-satisfying environment  ( ) Potential hazard in safety/hygiene  ( ) Not listed above_________________________________  [After answering this question, jump to C14.1] | Not used. |
| **C14.1** | C45.4 **single-choice** The service cost about _______Chinese Yuan every month (including childcare and food cost).  □≤1000 □~3000 □~5000 □~8000 □~10 000 □>10 000  [After answering this question, jump to M40] | Not used. |
| **C11.2** | C45.1 **single-choice** Which kind of center-based childcare service are you are planning to use?  □Public-owned □Private-owned □Owned by the parents’ employer  □Institutions providing early childhood education □Have no clue  [After answering this question, jump to C12.2] | Not used. |
| **C12.2** | C45.2 **ranking** Please rank the following aspects you value in choosing center-based childcare center:  ( ) High childcare fees  ( ) Suboptimal/lower than expected childcare concept  ( ) Poor quality of the staff  ( ) Far distance  ( ) Less-satisfying environment  ( ) Potential hazard in safety/hygiene  ( ) Not listed above_________________________________  [After answering this question, jump to C14.2] | Not used. |
| **C14.2** | C45.3 **single-choice** Your expected cost on this service is _______Chinese Yuan every month (including childcare and food cost).  □≤1000 □~3000 □~5000 □~8000 □~10 000 □>10 000  [After answering this question, jump to M40] | Not used. |
| **C12.3** | C45.1 **ranking** Please rank the following reasons for your unwillingness in using center-based childcare services:  ( ) We have plenty of childcare support  ( ) The cost of center-based childcare service is too high  ( ) There is no such service nearby  ( ) Worried about physical safety and health for the child  ( ) Not listed above ________________________  [After answering this question, jump to M40] | Not used. |
| **M40** | C46. **multiple-choice** In the last month, your child has participated in parent-child activities provided by the government for free/ at a lower price in the following places:  □ Infant and toddlers’ station/home  □ Community culture dissemination and exhibition center  □ Maternal and children’s hospitals  □ Early childhood development center  □ Community-based medical center  □ Else_________________________________  □ None, but I know where I can bring my child to engage in such activities  □ None, there is no such places for the afore-mentioned activities | Not used. |
| **M41** | C47. **single-choice** In the last month, you have spent _______Chinese Yuan in total for those activities.  □None □~100 □~200 □~500 □~1000 □>1000 | Not used. |
| **M42** | C48. **ranking** Please rank the following areas you feel confused in raising the child under 3 years’ old:  ( ) Sleep ( ) Feeding and growth ( ) Personality formation ( ) Emotion management ( ) Behavior problems  ( ) Language development ( ) Intelligence development ( ) Play ( ) Else  ( ) I have no confusions [This option is mutually exclusive with all the above options] | Not used. |
|  | **This is the end of the survey, thank you for your time!**  [The survey ends here] |  |

^a^All questions were asked orderly without randomization; words in blue denoting logic jumps between items, or explanation of certain options, or the code of each group for the corresponding variable that were re-classified in analysis.

^b^Numbers started with S are questions used for targeted respondents screening; numbers started with M are mandatory questions that all respondents must answer; numbers started with C are conditional must-answer questions that will only pop out when certain options are chosen by the respondent in the previous question.
