# Supplementary material for: Will a government subsidy increase couples’ further fertility intentions? A real-world study from a large-scale online survey in Eastern China
Source: Hum Reprod Open. 2024 Sep 17;2024(4):hoae055. doi: 10.1093/hropen/hoae055 (PMC11484797; doi:10.1093/hropen/hoae055)
Supplement: hoae055_Supplementary_Data [file hoae055_supplementary_data.zip › HRO-24-0170-R2-SuppTables1-3 .docx]

**Supplementary Table List**

**Supplementary Table S1.** Estimation of the potential impact of the subsidy policy on fertility rate in our study population

**Supplementary Table S2.** Estimation of the potential cost of the subsidy policy at provincial-scale

**Supplementary Table S3.** Factors associated with positive further fertility intention and positive intention change by current child number

**Supplementary Table S1. Estimation of the potential impact of the subsidy policy on fertility rate in our study population**^a^

| **Study population** | **Maternal age, years** | **Total** | **one-child family** | **two-child family** | **Expected births in 1 year^b^** | **Age-specific fertility rate, ‰^c^** | **Adjusted total fertility rate, ‰^d^** | **Cumulative change of fertility rate due to subsidy, %^e^** | **Cumulative total fertility rate, ‰^f^** |
| --- | --- | --- | --- | --- | --- | --- | --- | --- | --- |
| **Basic information** | 20-24 | 4541 | 3321 | 1220 |  |  |  |  |  |
|  | 25-29 | 45 724 | 32 272 | 13 452 |  |  |  |  |  |
|  | 30-34 | 63 092 | 30 191 | 32 901 |  |  |  |  |  |
|  | 35-39 | 24 902 | 5934 | 18 968 |  |  |  |  |  |
|  | 40-44 | 6634 | 1283 | 5351 |  |  |  |  |  |
|  | Sum | 144 893 | 73 001 | 71 892 |  |  |  |  |  |
| **Setting (1): all intentions, positive or negative, were fulfilled** | | | | | | | | | |
| **Positive FFI before learning about the subsidy policy** | 20-24 | 4541 | 1100 | 188 | 429 | 94.55 |  |  |  |
|  | 25-29 | 45 724 | 8589 | 1045 | 3211 | 70.23 |  |  |  |
|  | 30-34 | 63 092 | 6633 | 1815 | 2816 | 44.63 |  |  |  |
|  | 35-39 | 24 902 | 1048 | 985 | 678 | 27.21 |  |  |  |
|  | 40-44 | 6634 | 120 | 319 | 146 | 22.06 |  |  |  |
|  | Sum | 144 893 | 17 490 | 4352 | 7281 | 258.68 | 1293.42 |  |  |
| **Persistent negative FFI after learning about the subsidy** | 20-24 | 4541 | 1863 | 899 |  |  |  |  |  |
|  | 25-29 | 45 724 | 20 892 | 11 094 |  |  |  |  |  |
|  | 30-34 | 63 092 | 21 310 | 28 309 |  |  |  |  |  |
|  | 35-39 | 24 902 | 4491 | 16 260 |  |  |  |  |  |
|  | 40-44 | 6634 | 1074 | 4571 |  |  |  |  |  |
|  | Sum | 144 893 | 49 630 | 61 133 |  |  |  |  |  |
| **Positive FFI after learning about the subsidy policy** | 20-24 | 4541 | 358 | 133 | 164 | 36.04 |  |  |  |
|  | 25-29 | 45 724 | 2791 | 1313 | 1368 | 29.92 |  |  |  |
|  | 30-34 | 63 092 | 2248 | 2777 | 1675 | 26.55 |  |  |  |
|  | 35-39 | 24 902 | 395 | 1723 | 706 | 28.35 |  |  |  |
|  | 40-44 | 6634 | 89 | 461 | 183 | 27.64 |  |  |  |
|  | Sum | 144 893 | 5881 | 6407 | 4096 | 148.50 | 742.48 | 57.4 | 2035.90 |
| **Setting (2): 50% of positive intentions & 5% of negative intentions were fulfilled** | | | | | | | | | |
| **Positive FFI before learning about the subsidy policy** | 20-24 | 4541 | 1100 | 188 | 215 | 47.27 |  |  |  |
|  | 25-29 | 45 724 | 8589 | 1045 | 1606 | 35.12 |  |  |  |
|  | 30-34 | 63 092 | 6633 | 1815 | 1408 | 22.32 |  |  |  |
|  | 35-39 | 24 902 | 1048 | 985 | 339 | 13.61 |  |  |  |
|  | 40-44 | 6634 | 120 | 319 | 73 | 11.03 |  |  |  |
|  | Sum | 144 893 | 17 490 | 4352 | 3640 | 129.34 | 646.71 |  |  |
| **Negative FFI before learning about the subsidy policy** | 20-24 | 4541 | 2221 | 1032 | 54 | 11.94 |  |  |  |
|  | 25-29 | 45 724 | 23 683 | 12 407 | 602 | 13.16 |  |  |  |
|  | 30-34 | 63 092 | 23 558 | 31 086 | 911 | 14.44 |  |  |  |
|  | 35-39 | 24 902 | 4886 | 17 983 | 381 | 15.31 |  |  |  |
|  | 40-44 | 6634 | 1163 | 5032 | 103 | 15.56 |  |  |  |
|  | Sum | 144 893 | 55 511 | 67 540 | 2051 | 70.40 | 352.00 |  | 998.70 |
| **Persistent negative FFI after learning about the subsidy policy** | 20-24 | 4541 | 1863 | 899 | 46 | 10.14 |  |  |  |
|  | 25-29 | 45 724 | 20 892 | 11 094 | 533 | 11.66 |  |  |  |
|  | 30-34 | 63 092 | 21 310 | 28 309 | 827 | 13.11 |  |  |  |
|  | 35-39 | 24 902 | 4491 | 16 260 | 346 | 13.89 |  |  |  |
|  | 40-44 | 6634 | 1074 | 4571 | 94 | 14.18 |  |  |  |
|  | Sum | 144 893 | 49 630 | 61 133 | 1846 | 62.97 | 314.87 |  |  |
| **Positive FFI after learning about the subsidy policy** | 20-24 | 4541 | 358 | 133 | 82 | 18.02 |  |  |  |
|  | 25-29 | 45 724 | 2791 | 1313 | 684 | 14.96 |  |  |  |
|  | 30-34 | 63 092 | 2248 | 2777 | 838 | 13.27 |  |  |  |
|  | 35-39 | 24 902 | 395 | 1723 | 353 | 14.18 |  |  |  |
|  | 40-44 | 6634 | 89 | 461 | 92 | 13.82 |  |  |  |
|  | Sum | 144 893 | 5881 | 6407 | 2048 | 74.25 | 371.24 | 37.2 | 1332.82 |
| **FFI exclusively contributed by subsidy** | 20-24 | 4541 |  |  | 74 | 16.22 |  |  |  |
|  | 25-29 | 45 724 |  |  | 616 | 13.46 |  |  |  |
|  | 30-34 | 63 092 |  |  | 754 | 11.95 |  |  |  |
|  | 35-39 | 24 902 |  |  | 318 | 12.76 |  |  |  |
|  | 40-44 | 6634 |  |  | 83 | 12.44 |  |  |  |
|  | Sum | 144 893 |  |  | 1843 | 66.82 | 334.12 | 33.5 |  |

FFI, further fertility intention.

^a^Estimations were based on findings in the present study. Two settings were considered: 1) all FFI was fully realized in 3 years; 2) only 50% of positive FFI and 5% of negative FFI eventually deliver a live birth in 3 years. Before estimation, we assumed that 1) all/half (depends on the setting) positive FFI ended up with 1 healthy singleton in 3 years at a constant rate; 2) births contributed by families with all children ≥3 years, or already having ≥3 children, or with maternal age <20 or ≥45 years were not considered in estimations.

^b^The sum of births in one- and two- child families in 3 years divided by 3.

^c^Fertility rate in each specific age groups per 1000 women.

^d^The sum of age-specific fertility rate multiply by 5 years’ age-group intervals.

^e^The percentage of the adjusted total fertility rate exclusively contributed by the subsidy policy to the adjusted total fertility rate before subsidy.

^f^The sum of the adjusted total fertility rate exclusively contributed by the subsidy policy and the original total fertility rate before subsidy.

**Supplementary Table S2. Estimation of the potential cost of the subsidy policy at provincial-scale level**^a^

| **Data source** | **Indicators** | **One-child families** | **Two-child families** | **Total** |
| --- | --- | --- | --- | --- |
| **Governmental report** | Children <3 years during 2019-2021, n | 697 343 | 588 430 |  |
|  | Actual birth number in 2022, n |  |  | 412 000 |
|  | General Public Budget revenue in total in 2022, million CNY (million Euros) |  |  | 803.9(113.5) |
| **Results from our study** | Percentage of positive FFI before learning about the subsidy policy, % | 24.0 | 6.1 |  |
|  | Percentage of positive FFI after learning about the subsidy, % | 8.0 | 8.9 |  |
|  | Percentage of persistent negative FFI after learning about the subsidy policy, % | 68.0 | 85.0 |  |
| **Setting (1) Yearly estimations** | Births from respondents with positive FFI before learning about the subsidy policy, n | 55 787 | 11 965 | 67 752 |
|  | Births from respondents with positive FFI after subsidy, n | 18 596 | 17 457 | 36 053 |
|  | Births from respondents with persistent negative FFI after learning about the subsidy policy, n | 0 | 0 | 0 |
|  | Total number of births after subsidy, n | 74 383 | 29 422 | 103 805 |
|  | Cost before the subsidy policy, million CNY (million Euros)^b^ | 669.4(94.5) | 143.6(20.3) | 813.0(114.8) |
|  | Increased cost due to the subsidy policy, million CNY (million Euros)^b^ | 223.1(31.5) | 209.5(29.6) | 432.6(61.1) |
|  | Total cost after the subsidy policy, million CNY (million Euros)^b^ | 892.6(126.0) | 353.1(49.9) | 1245.7(175.9) |
|  | Percentage of cost due to subsidy relative to the cost before the subsidy policy, % | 33.3 | 145.9 | 53.2 |
|  | Percentage of estimated annual cost in General Public Budget Revenue, ‰ | 1.11 | 0.44 | 1.55 |
|  | Times of cost covering all births relative to annual General Public Budget Revenue^c^ | 6.4 | 6.4 | 6.7 |
| **Setting (2) Yearly estimations** | Births from respondents with negative FFI before learning about the subsidy policy, n | 8833 | 9209 | 18 042 |
|  | Births from respondents with positive FFI before learning about the subsidy policy, n | 27 894 | 5982 | 33 876 |
|  | Births from respondents with positive FFI after subsidy, n | 9298 | 8728 | 18 026 |
|  | Births from respondents with persistent negative FFI after learning about the subsidy policy, n | 7903 | 8336 | 16 239 |
|  | Total number of births after subsidy, n | 45 095 | 23 047 | 68 142 |
|  | Cost before the subsidy policy, million CNY (million Euros)^b^ | 440.7(62.2) | 182.3(25.7) | 623.0(88.0) |
|  | Increased cost due to the subsidy policy, million CNY (million Euros)^b^ | 100.4(14.2) | 94.3(13.3) | 194.7(27.5) |
|  | Total cost after the subsidy policy, million CNY (million Euros)^b^ | 541.1(76.4) | 276.6(39.1) | 817.7(115.5) |
|  | Percentage of cost due to subsidy relative to the cost before the subsidy policy, % | 22.8 | 51.7 | 31.2 |
|  | Percentage of estimated annual cost in General Public Budget Revenue, ‰ | 0.67 | 0.34 | 1.02 |
|  | Times of cost covering all births relative to annual General Public Budget Revenue^c^ | 6.3 | 6.3 | 6.4 |

CNY, Chinese Yuan (1 CNY=0.1412 Euros in 2022); FFI, further fertility intention.

^a^Estimations were based on based on findings in the present study and public-available data. Two settings were considered: 1) all FFI was fully realized in 3 years; 2) only 50% of positive FFI and 5% of negative FFI eventually deliver a live birth in 3 years. Before estimation, we assumed that1) all/half (depends on the setting) positive FFI ended up with 1 healthy singleton in 3 years at a constant rate; 2) births contributed by families with all children ≥3 years, or already having ≥3 children, or with maternal age <20 or ≥45 years were not considered in estimations. Also, all estimations regarding cost were based on the ￥1000/month subsidy policy for each additional birth.

^b^The subsidy covers only targeted to families already having at least 1 child.

^c^The subsidy covers both actual births in 2022 regardless of parities and potential increased births due to the subsidy policy.

**Supplementary Table S3. Factors associated with positive further fertility intention and positive intention change by current child number**^a,b^

| **Characteristics** | **Positive FFI before subsidy** | | **Positive FFI change after subsidy** | |
| --- | --- | --- | --- | --- |
|  | **One-child families** | **Two-child families** | **One-child families** | **Two-child families** |
|  | **OR (95% CI)** | **OR (95% CI)** | **OR (95% CI)** | **OR (95% CI)** |
| **Sex of the respondents (ref=Male)** | 0.81(0.77-0.85) | 0.67(0.62-0.73) | 0.74(0.69-0.79) | 0.69(0.64-0.74) |
| **Migration status (ref=Local)** |  |  |  |  |
| In-provincial migrants | 1.09(1.00-1.18) | 0.98(0.84-1.15) | / | 0.82(0.71-0.94) |
| Inter-provincial migrants | 1.38(1.28-1.49) | 1.19(1.07-1.31) | / | 0.85(0.78-0.93) |
| **Residence (ref=Urban)** |  |  |  |  |
| More developed rural | 1.44(1.38-1.50) | 1.09(1.01-1.17) | 1.17(1.10-1.25) | 0.96(0.91-1.02) |
| Under-developed rural | 1.66(1.58-1.73) | 1.26(1.16-1.37) | 1.30(1.21-1.40) | 1.11(1.04-1.19) |
| **Maternal age, years (ref=20-24)** |  |  |  |  |
| 25-29 | 0.85(0.78-0.92) | 0.63(0.53-0.75) | 0.80(0.71-0.91) | 0.84(0.69-1.02) |
| 30-34 | 0.72(0.66-0.78) | 0.54(0.45-0.64) | 0.67(0.59-0.77) | 0.76(0.62-0.92) |
| 35-39 | 0.55(0.50-0.61) | 0.52(0.44-0.63) | 0.55(0.47-0.64) | 0.87(0.71-1.06) |
| 40-44 | 0.26(0.21-0.32) | 0.61(0.50-0.75) | 0.49(0.38-0.63) | 0.88(0.71-1.09) |
| **Maternal education level (ref=≤Elementary school )** |  |  |  |  |
| High school | 0.94(0.86-1.02) | 0.73(0.66-0.80) | 0.99(0.87-1.12) | / |
| Junior college | 0.76(0.70-0.82) | 0.62(0.56-0.69) | 0.89(0.79-1.00) | / |
| University or higher | 0.74(0.68-0.80) | 0.58(0.52-0.64) | 0.83(0.74-0.94) | / |
| **Age of the youngest child, months** | / | 0.97(0.97-0.98) | / | 0.97(0.96-0.97) |
| **Sex of the youngest child (ref=Boy)** | 1.31(1.27-1.36) | 1.43(1.35-1.53) | 1.10(1.05-1.17) | 1.06(1.01-1.12) |
| **Household income, CNY/year (ref=≤100 000)** |  |  |  |  |
| 100 001-200 000 | 0.99(0.95-1.03) | 0.85(0.79-0.92) | 0.87(0.81-0.93) | 0.85(0.80-0.91) |
| 200 001-300 000 | 1.02(0.96-1.09) | 0.79(0.70-0.88) | 0.69(0.62-0.76) | 0.73(0.67-0.80) |
| ＞300 000 | 1.35(1.26-1.45) | 0.98(0.87-1.11) | 0.70(0.62-0.79) | 0.61(0.55-0.68) |
| **Financial support from grand-parents, CNY/year (ref=None)** |  |  |  |  |
| ≤50 000 | 1.17(1.13-1.22) | 0.99(0.92-1.07) | / | / |
| 50 001-100 000 | 1.46(1.38-1.55) | 1.27(1.13-1.41) | / | / |
| ＞100 000 | 1.75(1.63-1.88) | 1.76(1.55-1.98) | / | / |
| **House loan/ rent, CNY/year (ref=None)** |  |  |  |  |
| ≤30 000 | 0.83(0.78-0.88) | / | 1.13(1.02-1.24) | 1.20(1.09-1.32) |
| 30 001-60 000 | 0.77(0.74-0.81) | / | 1.17(1.08-1.26) | 1.30(1.20-1.40) |
| 60 001-120 000 | 0.79(0.75-0.83) | / | 1.24(1.15-1.34) | 1.30(1.20-1.41) |
| ＞120 000 | 0.91(0.85-0.97) | / | 1.28(1.15-1.42) | 1.27(1.16-1.40) |
| **Expenses on children's education, CNY/year (ref=≤5 000)** |  |  |  |  |
| 5 001-10 000 | 0.91(0.86-0.96) | 0.85(0.78-0.93) | 1.01(0.92-1.10) | 1.01(0.94-1.09) |
| 10 001-25 000 | 0.81(0.77-0.86) | 0.81(0.75-0.89) | 0.89(0.82-0.97) | 0.92(0.85-0.99) |
| ＞25 000 | 0.73(0.68-0.78) | 0.90(0.82-0.99) | 0.80(0.72-0.88) | 0.98(0.90-1.06) |
| **Childcare by the mother, hours/day (ref=0)** |  |  |  |  |
| 0.1-1.9 | 0.87(0.73-1.04) | 0.97(0.74-1.26) | / | / |
| 2-5.9 | 0.85(0.72-1.01) | 0.74(0.58-0.96) | / | / |
| ≥6.0 | 0.83(0.70-0.97) | 0.68(0.53-0.86) | / | / |
| **Childcare by the father, hours/day (ref=0)** |  |  |  |  |
| 0.1-1.9 | 1.23(1.13-1.34) | 1.04(0.90-1.19) | / | 1.08(0.97-1.21) |
| 2-5.9 | 1.33(1.22-1.45) | 1.21(1.05-1.39) | / | 1.10(0.99-1.23) |
| ≥6.0 | 1.39(1.27-1.52) | 1.26(1.08-1.46) | / | 1.23(1.10-1.38) |
| **Childcare by the grandparents, hours/day (ref=0)** |  |  |  |  |
| 0.1-1.9 | 1.16(1.09-1.24) | 1.09(0.99-1.20) | / | / |
| 2-5.9 | 1.31(1.23-1.39) | 1.08(0.98-1.19) | / | / |
| ≥6.0 | 1.29(1.22-1.36) | 0.86(0.79-0.95) | / | / |

CI, confidence intervals; CNY, Chinese Yuan (1 CNY=0.1412 Euros in 2022); FFI, further fertility intention; OR, odds ratio. Step-wise logistic regression models were used in understanding associated factors of positive FFI in the beginning and positive FFI change from “No” to “Yes” after learning about the subsidy policy.

^a^Only factors included in the final model of stepwise logistic regressions were presented.

^b^In the models, “Yes” was treated as the event and “No” as the reference for each outcome, i.e., OR>1 denoting a higher likelihood of positive FFI/positive FFI change relative to the reference group.
